# Considering Gender Differences in Portion Sizes to Improve the Accuracy of Nutrient Intakes from A Food Frequency Questionnaire

**DOI:** 10.3390/nu11071449

**Published:** 2019-06-26

**Authors:** Minji Kang, Song-Yi Park, Carol J. Boushey, Lynne R. Wilkens, Loïc Le Marchand, Laurence N. Kolonel, Suzanne P. Murphy, Hee-Young Paik

**Affiliations:** 1 Center for Gendered Innovations in Science and Technology Research (GISTeR), Korea Federation of Women’s Science & Technology Associations, Seoul 06130, Korea; 2 Cancer Epidemiology Program, University of Hawaii Cancer Center, Honolulu, HI 96813, USA

**Keywords:** food frequency questionnaire, Multiethnic Cohort Study, portion size, gender

## Abstract

The aim of this study was to examine whether using gender specific-portion size (GS-PS) improves the accuracy of nutrient intake assessment by a quantitative food frequency questionnaire (QFFQ). For GS-PS quantification, a gram amount was assigned to each PS category for each food item for men and women separately using data from three 24 h dietary recalls (24HDRs) in a calibration study of the Multiethnic Cohort (men = 1141, women = 1150). Nutrient intakes were calculated from the QFFQ using the original-PS and the GS-PS, and were compared with 24HDRs. When intakes of energy and 15 nutrients were compared, absolute intakes calculated using the GS-PS were closer to intake levels of 24HDRs in both men and women. Using GS-PS did not affect intakes expressed as nutrient density or correlations between 24HDRs and the QFFQ. The current findings indicate that considering gender in PS determination can increase the accuracy of intake assessment by QFFQ for absolute nutrient intakes, but not for nutrient densities.

## 1. Introduction

In large-scale epidemiological studies, food frequency questionnaires (FFQs) are the most frequently used tool to assess an individual’s usual intake over the past several months to years, typically for one year [1,2]. However, intake estimated from FFQs may differ from the true intake for several reasons, including difficulties in long-term recall, influence of psychological factors such as social desirability, and difficulties in estimation of the consumption frequencies and average portion sizes (PSs) of each food item [3]. Therefore, efforts to improve the quality of dietary assessment data are essential to improving the estimation of diet–disease relationships. Using the characteristics of the target population to assign food items more accurate PS in FFQs has the potential to improve the quality of dietary intake data, as the estimation of the usual PS is one of the sources of measurement error.

Dietary intakes assessed by dietary survey can be influenced by many factors, including age, gender, day of the week, interviewers, and cultural diversity [4,5,6,7]. However, among the factors examined, Beaton et al. reported that gender was a major contributing factor in the variance of nutrient intake [4]. In addition, differences in food consumption between men and women have been observed in several studies. For many, but not all single foods or food groups examined, men reported larger PSs on average than women [8,9,10,11,12,13]. However, gender differences in food intakes are rarely considered in determining PSs of FFQs [14]. It is not known how specifying PSs by gender would affect nutrient intake assessment in cohort studies using QFFQs. 

The Multiethnic Cohort Study (MEC) is a population-based cohort in Hawaii and Los Angeles established to study diet and cancer associations. In the MEC, a QFFQ was used to collect dietary intake data. More than 180 food items were listed in the QFFQ with PS options, which were based on typical serving sizes for each item as reflected in three-day measured food records in a multiethnic population [15,16]. Our previous studies showed that gender differences exist in amounts of foods consumed by participants who chose the same PS categories, but ratios of food amounts consumed in the smallest or largest PS to the medium PS were similar in men and women [17,18]. There has been no study examining whether errors in nutrient intake assessment by QFFQ can be reduced by assigning PS amounts specific to each gender.

This study aims to find suitable methods to determine gender specific-PS (GS-PS) for items on the QFFQ utilizing data from three 24-h dietary recalls (24HDRs) collected during a calibration study in the MEC, and to examine whether using GS-PS improves the accuracy of nutrient intake assessment from the QFFQ, compared with using common PS amounts.

## 2. Methods

### 2.1. Study Participants

The MEC was established in 1993–1996 to study diet and cancer associations among different ethnic groups [15]. The cohort consisted of 215,251 men and women (age 45–75 years at baseline) living in Hawaii and Los Angeles who were primarily of five major race/ethnicities: African American, Native Hawaiian, Japanese American, Latino, and non-Hispanic white [15]. Participants completed a self-administered 26-page questionnaire that included information on demographic factors, a QFFQ, lifestyle behaviors, history of medical conditions, use of medications, family history of cancer, and a reproductive history for women [15].

The current study used dietary data from a calibration study in the MEC, which was conducted in 1994–1997, to evaluate the performance of the QFFQ for each ethnic-sex group. Details of the calibration study have been described previously [19]. Briefly, for each ethnic-sex subgroup, a random sample of approximately 260 people was targeted for inclusion from the lists of cohort members [19]. Three unannounced 24HDRs were collected by telephone about one month apart by registered dietitians who were trained in eliciting 24HDR information. A repeat QFFQ was administered approximately 4–6 weeks after the three 24HDRs were completed [19]. This repeat QFFQ was identical to the baseline QFFQ, and covered the reference period of the 24HDRs. The Institutional Review Boards at the University of Hawaii and the University of Southern California approved the study protocol, and all participants provided written informed consent. The final sample for the current analysis included 2291 participants who completed three 24HDRs and the repeat QFFQ. For the purposes of this study, the QFFQ refers to the repeat QFFQ.

### 2.2. Dietary Data Collection: QFFQ and 24HDRs

The QFFQ was developed based on three-day measured food records from approximately 60 men and women, 45 to 75 years of age, from each of the five main ethnic groups [15]. The food item for the QFFQ was based on a minimum set that contributed at least 85 percent to the total intake of important nutrients (e.g., fat, dietary fiber, vitamin A, and vitamin C) within each ethnic group. The final questionnaire list actually accounts for much more than 85 percent of the intake of the major nutrients, as it contains foods that contribute to only one ethnic group [15]. More than 180 food items were listed in the QFFQ with eight frequency categories for foods and nine for beverages, together with a PS option for 163 items [15]. Participants could choose the size that most often typified their usual portion of each food item, with three PS options for 151 items, four options for 7 items (alcoholic beverages and sodas), and two options for 5 items (bread spreads) [15]. The PS options were based on typical serving sizes for each items as reflected in the distribution from the three-day measured food records, and converted to interpretable quantities, such as cups [15]. The distribution for many foods had three common peaks; therefore, choices of three portion sizes were provided in the QFFQ based broadly on the common amounts [5,6,15,16]. For example, mixed dishes were given in specific amounts (e.g., half a cup, 1 cup, 2 cups, and so on) based on the most commonly consumed amounts in grams within the ranges of the three peaks. For foods that did not exhibit three common peaks, three portion sizes were defined based on percentiles. An exception was countable items, such as eggs, which were given in natural units (e.g., 1, 2, 3). Four serving size options were provided for beverage items on the QFFQ. However, these are not small, medium, and large concepts, but rather multiples of a standard portion size, such as half, 1, 2, and 3 cans of soda. Two serving sizes were provided for certain bread spreads, such as mayonnaise and peanut butter, labelled as “spread thin” or “spread thick”. This was to account for the fact that these food items could contribute substantially to energy or macronutrient intakes. As an additional aid to quantification, photographs illustrating three PSs for representative food items were provided at the head of several pages of the questionnaire [15]. 

For the calibration study, three unannounced 24HDRs (of the previous day’s diet) were collected by telephone by registered dietitians. Portion sizes on the 24HDRs were based on customary household measures, such as cups, teaspoons, or ounces. A set of measuring cups and spoons were provided to each calibration study participant to assist them with quantification of serving size. Intakes from the QFFQ and 24HDRs were calculated using the food composition tables developed and maintained at the University of Hawaii Cancer Center for use in the MEC [15,19,20].

### 2.3. Gender Specific-Portion Size (GS-PS) Quantification

Each food item on the QFFQ typically represented several component foods. For example, the “whole wheat or rye bread” item of the QFFQ contained five component foods: “whole wheat bread”, “light rye bread”, “dark rye bread”, “pita whole wheat bread”, and “wheat bran bread”. Individual foods reported from 24HDRs were matched with component foods that comprised each food item in the QFFQ. Average amounts per eating occasion for any of these component foods were computed from the 24HDRs. This was generally calculated as the average daily intake; however, when a participant consumed a specific component more than once in a day, the daily amount was divided by the number of times eaten. These data were then used to assign a gram amount to each of the PS categories (A, the smallest; B; and C or D, the largest) for men and women separately. The largest PS category, D, was used for only seven beverage items. The median intake amount of the matched foods reported in 24HDRs by the participants who chose a specific PS category of the QFFQ item served as a reasonable GS-PS of that portion. For example, we identified the amount of each of the five component foods for the “whole wheat or rye bread” item on the QFFQ that were reported in 24HDRs by each individual woman who chose PS category A for that item. Then, the median value of the average amount per eating occasion for the identified foods was calculated and used as the gram amount for women for PS category A for this food item on the QFFQ. This principle required that a reasonable number of participants chose each individual PS category of an item on the QFFQ and also consumed the food components within the item in the three 24HDRs that were collected. 

For this study, the preference was to assign the median value for the relevant foods from the 24HDRs for each QFFQ food item and PS. However, for many cases, especially for categories A and C, the number of participants was not sufficient to determine median values. Different rules were applied to quantify GS-PS for each of the food items by PS category depending on the available data. The four rules that were used in determining GS-PS, ordered by priority, are described below.

Rule I. Using gender specific median values from 24HDRs data: For each PS category (A, B, C, D) of items on the QFFQ that were selected by five or more participants and consumed by five or more participants in 24HDRs, median values from 24HDRs were used for the PS of that category of the item. In Rule I, we used the gender specific median values only when the values were in the expected order. For example, the PS of category A was determined using gender specific median values for an item only if the median value of A was smaller than B.

Rule II. Imputed values based on the general ratios of median values of categories A and C to B (A/B, C/B): For PS categories that were not consumed by five or more participants either in the QFFQ or 24HDRs (usually A, C, or D PS categories), the PS of the category was determined by applying ratios to the computed B portion (A/B, C/B). Mean ratios A/B and C/B used in this study were 0.71 and 1.45 for men, and 0.71 and 1.44 for women, respectively. Details of the method of obtaining the ratios have been published separately [18].

Rule III. Adapting the value of another PS category of the food item (usually category B): This rule was applied when a sufficient number of people consumed the item, and no clear distinction existed between participants who chose two or three PS categories. For example, the same GS-PS of “macaroni or potato salad” for men was assigned to both PS A and B; this is because 1) median values derived from 24HDRs of PSs A and B for this item among men were the same, and 2) the number of reported people from the 24HDRs was sufficient for both PSs A and B.

Rule IV. Using the original-PS: For some items, insufficient information was available to change the PS by any of the above rules. Usually, food items were consumed by less than five participants in all PS categories from the 24HDRs, and it was not possible to determine median values or examine distributions by gender. For these items, the original-PSs of the QFFQ were used.

### 2.4. Statistical Analysis

Daily nutrient intakes were estimated from the QFFQ using the original-PS (original-PS QFFQ) and the GS-PS (GS-PS QFFQ). Average nutrient intakes over the three 24HDRs were calculated with weighting according to the day of week (by 5/7 for weekday and 2/7 for weekend), as weekends contribute less to the average than weekdays. Mean daily nutrient intakes from the QFFQs (original-PS QFFQ or GS-PS QFFQ) were compared to those from weighted 24HDRs by paired t-tests for men and women separately. Pearson correlation coefficients between the QFFQs (original-PS or GS-PS QFFQ) and 24HDRs were calculated. All correlations were based on log transformed nutrient values. To account for the fact that there was substantial within-person variability based on only three recall values rather than a large number, the correlations were adjusted using a reliability factor defined as the ratio of between-person to total variance in the 24HDRs [19]. Average daily nutrient intakes and correlations were also computed in 200 bootstrap samples, which were repeatedly drawn from the original sample with replacement. This will provide a random mixing of assignment patterns to GS-PS, which should remove some of the bias caused by the dependence of the PS assignment process on the 24HDR values.

The degree of misclassification between nutrients computed from the original-PS and the GS-PS QFFQ was further assessed by comparing quintile distributions, as associations for extreme intakes are often reported in epidemiologic studies. In particular, separately for men and women, absolute nutrient intakes were classified into a quintile for the QFFQ with original-PS, and classified into a quintile for the QFFQ with GS-PS. For each nutrient, participants in the lowest and the highest quintiles from the QFFQ with the original-PS were compared to the quintiles they belonged to using intakes obtained from the QFFQ with GS-PS. A similar analysis was also performed for intakes expressed as nutrient densities. Data were analyzed using SAS statistical software version 9.4 (SAS Institute Inc., Cary, NC, USA) [21], and the level of significance was set at *p* < 0.05.

## 3. Results

### 3.1. Participant Characteristics

Background characteristics of participants who completed the QFFQ and three 24HDRs are presented in Table 1. Among the 2291 participants included in this analysis, 50% were women. Race/ethnicity and age group distributions were well balanced between men and women by design. Men tended to have higher education levels and were more often former or current smokers when compared with women (*p* < 0.05). Women were less likely to be overweight (body mass index, BMI 25–30 kg/m^2^), and more likely to be obese (BMI ≥ 30 kg/m^2^) than men.

### 3.2. Gender Specific-Portion Size (GS-PS)

The number of foods and beverage items that used each rule to assign GS-PS by PS category and gender is presented in Table 2. In all PS categories, rule I, using gender specific median values from the 24HDRs, was most frequently used in both men and women, with about two-thirds of all categories being determined by rule I. This was especially true for category B, where about 90% of the GS-PS was determined by rule I. For men, rule I was used less for category A (the smallest PS), but more for category C (usually the largest PS) compared with women. Rule II, using imputed values based on ratios, was used more in men, especially for category A, when compared with women. However, there was no significant difference in the distribution of rules applied between men and women (chi-square test, *p* = 0.2254)

When the gram amounts assigned to the newly assigned GS-PS were compared to those for the original-PS of the QFFQ used in the MEC, about 30% of GS-PS were in the range of 90%–110% of the original-PS in both men and women (Table 3). The GS-PS was more likely to be smaller than the original-PS for women than for men (50.5% vs. 42.4%), while the GS-PS was more likely to be larger than the original value for men than for women (25.7% vs. 19.0%), and these distributions were significantly different between men and women (*p* = 0.0136). For PS category A, the top three food items that had the largest differences between GS-PS and original-PS were as follows: for men, “beef steak or roast, veal, or lamb (GS-PS: 99 g vs. original-PS: 28 g)”; “ramen or saimin (341 vs. 120 g)”; and “poi (170 g vs. 60 g)”; for women, “fried fish (85 g vs. 28 g)”, “poi (180 g vs. 60 g)”, and “ramen or saimin (341 g vs. 120 g)”. For PS C, the top three food items that had the largest differences were as follows: for men, “popcorn (36 g vs. 165 g)”, “canned tunafish (50 g vs. 180 g)”, and “ham (40 g vs. 142 g)”; for women, “potato, corn, tortilla, or other chips (20 g vs. 105 g)”; “macaroni or potato salad (120 g vs. 450 g”; and “cookies, brownies, or fruit bars (30 g vs. 110 g)”. 

### 3.3. Utilizing GS-PS to Calculate Dietary Intake by QFFQ

**Figure 1:**
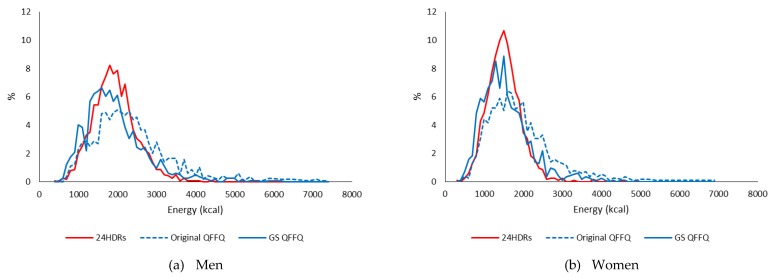
Distributions of energy intake estimated from 24 h dietary recalls (24HDRs) and those from quantitative food frequency questionnaire (QFFQ) using either an original-portion size or a gender specific (GS)-portion size in the calibration study of the Multiethnic Cohort. (**a**) men. (**b**) women.

Mean daily nutrient intakes calculated from the QFFQs (original-PS QFFQ and GS-PS QFFQ) in men and women are presented along with those from 24HDRs in Table 4. For absolute nutrient intakes, the values using the GS-PS were closer to the 24HDRs (the reference method) than the values using the original-PS in both men and women. However, for nutrient densities, the values using GS-PS and original-PS were similar. The distributions of energy intake obtained from the GS-PS QFFQ were closer to the distribution obtained from 24HDRs compared with the distribution from original-PS QFFQ in both men and women (Figure 1).

Table 5 shows the Pearson correlation coefficients between 24HDRs and the QFFQ using original-PS or GS-PS. For absolute nutrient intakes, the average correlation coefficients between the 24HDRs and the QFFQ using original-PS were 0.40 for both men and women. For the GS-PS QFFQ, the corresponding average correlation coefficients were 0.38 for both men and women. When nutrient intakes were expressed as nutrient densities, the average correlations were higher, at 0.61 between the 24HDRs and the original-PS QFFQ for both men and women, and 0.61 and 0.60 between the 24HDRs and the GS-PS QFFQ for men and women, respectively. Average estimates of multiple samples derived from bootstrapping were very close to those presented in Table 4 and Table 5. In particular, the correlation coefficients with 24HDRs in the bootstrap analysis were as follows: (i) absolute nutrient intake: original-PS: 0.40 for both men and women, and GS-PS: 0.38 for both men and women; (ii) nutrient densities: original-PS: 0.61 for both men and women, and GS-PS: 0.61 for men and 0.60 for women.

Correlation coefficients between nutrient intakes for the original-PS and the GS-PS were all above 0.95. Classification of participants into extreme quintiles by nutrient intake levels estimated with the original-PS QFFQ and the GS-PS QFFQ were compared (Supplementary Table S1). Among the participants assigned to the lowest quintile of energy intake by the original-PS QFFQ, 86.0% in men and 87.0% in women belonged to the lowest category by the GS-PS QFFQ. For those in the highest category, the percentages were 86.0% and 83.9% in men and women, respectively. Averaged across nutrients, the percent of participants belonging to the same lowest (88.0% in men and 87.3% in women) or highest quintile (88.0% in men and 86.7% in women) were similar for both genders. When nutrient density was used, the percentages increased only slightly.

## 4. Discussion

One of the goals of the present study was to explore a suitable method to determine GS-PS for items on a QFFQ. GS-PS as a single gram amount was assigned to each category of PS for each item in the QFFQ for men and women separately, using data from three days of 24HDRs. Four rules were applied in determining GS-PS depending on the availability of data to derive median value from 24HDRs among those who chose each category of food items on QFFQ. As about 68% of the GS-PS was determined by rule I, using gender specific median values from 24 HDRs, in both men and women, it is reasonable to assume the GS-PS values would reflect the actual consumption of the participants better than the original-PS, which was based on food records from a different population. Importantly, about 90% of the GS-PS for category B, which is the most commonly chosen category and is also utilized to set values by rules II & III, could be determined by rule I. Although the percent distribution of the four rules applied to determine GS-PS was not significantly different by gender, the gram amounts assigned to the GS-PS, as percentages of the original-PS values, were distributed differently between men and women.

Nutrient intakes estimated using the GS-PS were closer to the 24HDRs than the values estimated using the original-PS in both men and women for absolute nutrient intakes, but not for intakes expressed as nutrient densities. These results demonstrate that the absolute intake levels are influenced by adjusting PS by gender, but nutrient density and correlations are not affected significantly. Nöthlings and colleagues compared the use of fitted PSs with the use of predefined PSs using data from a validation study in the European Prospective Investigation into Cancer and Nutrition-Potsdam Study [22]. The fitted PSs were used to calculate an average daily intake of each food item consumed in the two 24HDRs and were summed separately for men and women and divided by the summed frequencies of consumption per day reported in the FFQ [22]. Absolute intakes of nutrients were closer to the 24HDRs after implementation of fitted PSs compared with predefined PSs. However, the effect on the ranking of study participants was only marginal and correlation coefficients using fitted PSs did not generally improve [22].

The correlations between the daily nutrient intake from 24HDRs and those from QFFQ either using original-PS or GS-PS were similar to each other and to those previously reported in the calibration study using original-PS in the MEC [19]. Intakes are often divided into quantiles in nutritional epidemiologic studies [23,24,25,26,27,28]. For instance, in a previous analysis of the MEC, a comparison between the highest and lowest categories indicated that dietary fiber intake was inversely associated with colorectal cancer risk in both sexes: Hazard Ratio (HR) = 0.73, 95% Confidence Interval (CI): 0.61–0.89 for highest versus lowest quintile in men and HR = 0.76, 95% CI: 0.62–0.91 in women [25]. When the classification of participants into quintiles by nutrient intake levels quantified with the QFFQ using original-PS and GS-PS were compared, 86.6 to 88.8% of participants were classified in the same lowest or highest quintile. Given the similar correlations with the 24HDRs for nutrient intakes calculated from the original-PS QFFQ and the GS-PS QFFQ, as well as the similar classifications of participants into extreme quintiles, it is possible that using the GS-PS QFFQ may not greatly change associations of nutrient intakes with disease outcomes. However, it is premature to predict how the use of GS-PS would affect the magnitude of such associations.

The strengths of the study include a large number of participants from a population-based multiethnic cohort, and use of a QFFQ that covered the reference period of the 24HDRs. Several limitations should be taken into consideration when interpreting the findings from this study. First, although food amounts from three 24HDRs were used as the reference values, 24HDRs are subject to measurement error inherent to all self-reported dietary assessment methods such as memory recall errors and social desirability [3,19,29]. We minimized concerns about within-person variance of the 24HDRs by using appropriate statistical analyses. Another possible limitation is that we used the 24HDRs not only to set the GS-PS, but also as a reference for comparison. To address this issue, daily nutrient intakes and correlations were computed in bootstrap samples, and the results did not change. Additionally, the study participants were middle-aged or older and resided in ethnically diverse populations, which likely influenced their diet choices; therefore, the findings may not apply to younger and more homogeneous populations.

## 5. Conclusions

In conclusion, the use of GS-PS contributed to the accuracy of dietary data from a QFFQ especially in absolute, but not in energy-adjusted intakes in both men and women. For nutritional epidemiological studies, which use dietary variables adjusted for energy intake, such as % energy or nutrient densities, using GS-PS may not be necessary. For studies using absolute intakes of nutrients, foods, food groups, or dietary patterns, GS-PS is likely to be beneficial. Further studies are warranted to investigate the impact of GS-PS on the relations between diet factors and disease outcomes.

## Figures and Tables

**Table 1:** Baseline characteristics of participants who completed the quantitative food frequency questionnaire and three 24 h dietary recalls in the calibration study of the Multiethnic Cohort.

	Men (*n* = 1141)	Women (*n* = 1150)	*p*-value ^1^
Race/ethnicity			
African American	168 (14.7)	197 (17.1)	0.4746
Native Hawaiian	213 (18.7)	217 (18.9)	
Japanese American	292 (25.6)	296 (25.7)	
Latino	168 (14.7)	150 (13.0)	
Non-Hispanic white	300 (26.3)	290 (25.2)	
Age			
45–54 years	283 (24.8)	324 (28.2)	0.0973
55–64 years	342 (30.0)	352 (30.6)	
65–75 years	516 (45.2)	474 (41.2)	
Education			
≤ High school graduate	388 (34.4)	449 (39.7)	0.0160
Vocational school/some college	357 (31.7)	352 (31.1)	
≥ College graduate	382 (33.9)	330 (29.2)	
Body mass index			
<25 kg/m^2^	491 (43.0)	590 (51.3)	<.0001
25–30 kg/m^2^	490 (42.9)	348 (30.3)	
≥30 kg/m^2^	160 (14.0)	212 (18.4)	
Smoking Status			
Never	369 (32.8)	628 (55.8)	<.0001
Former smoker	579 (51.5)	359 (31.9)	
Current smoker	177 (15.7)	139 (12.3)	

^1^*p*-values from chi-square test.

**Table 2:** Number (%) of food and beverage items by portion size categories on the quantitative food frequency questionnaire that used each rule to determine gender specific-portion sizes in the Multiethnic Cohort. 24HDRs—24 h dietary recalls.

Rule	Men					Women				
	Portion Size Categories				Overall	Portion Size Categories				Overall
	A	B	C	D		A	B	C	D	
I. Using gender specific median values from 24HDRs	79 (48.5)	147 (90.2)	104 (65.8)	6 (85.7)	336 (68.4)	107 (65.6)	145 (89.0)	83 (52.5)	3 (42.9)	338 (68.8)
II. Imputing values using ratios to median values	54 (33.1)	4 (2.5)	26 (16.5)	-	84 (17.1)	33 (20.2)	2 (1.2)	31 (19.6)	-	66 (13.4)
III. Adapting the values of other portion sizes	19 (11.7)	1 (0.6)	19 (12.0)	-	39 (7.9)	12 (7.4)	5 (3.1)	35 (22.2)	1 (14.3)	53 (10.8)
IV. Using the original-portion size	11 (6.7)	11 (6.7)	9 (5.7)	1 (14.3)	32 (6.5)	11 (6.7)	11 (6.7)	9 (5.7)	3 (42.9)	34 (6.9)
Total	163	163	158	7		163	163	158	7	

**Table 3:** Number (%) of food items in each portion size category by the ratio of the values of the newly determined gender specific-portion sizes relative to the values of the original-portion sizes (expressed as a percentage) of the Multiethnic Cohort quantitative food frequency questionnaire.^1.^

Original Portion Size Category	% of Gender Specific-Portion Size Values to Original-Portion Size Values						
	<50%	50%–70%	70%–90%	90%–110%	110%–130%	130%–150%	>150%
	Men						
A	1 (0.6)	6 (3.7)	12 (7.4)	50 (30.7)	16 (9.8)	26 (16.0)	52 (31.9)
B	10 (6.1)	27 (16.6)	32 (19.6)	68 (41.7)	21 (12.9)	2 (1.2)	3 (1.8)
C	31 (19.6)	50 (31.7)	35 (22.2)	36 (22.8)	6 (3.8)	-	-
D	2 (28.6)	-	2 (28.6)	3 (42.9)	-	-	-
Overall	44 (9.0)	83 (16.9)	81 (16.5)	157 (32.0)	43 (8.8)	28 (5.7)	55 (11.2)
	Women						
A	-	14 (8.6)	16 (9.8)	50 (30.7)	14 (8.6)	27 (16.6)	42 (25.8)
B	12 (7.4)	36 (22.1)	38 (23.3)	67 (41.1)	5 (3.1)	3 (1.8)	2 (1.2)
C	38 (24.1)	62 (39.2)	28 (17.7)	30 (19.0)	-	-	-
D	2 (28.6)	1 (14.3)	1 (14.3)	3 (42.9)	-	-	-
Overall	52 (10.6)	113 (23.0)	83 (16.9)	150 (30.5)	19 (3.9)	30 (6.1)	44 (9.0)

^1^ Distributions between men and women are significantly different by chi-square test (*p* = 0.0136).

**Table 4:** Daily nutrient intakes (absolute and as densities) estimated from 24 h dietary recalls (24HDRs) and from the quantitative food frequency questionnaire (QFFQ) using either an original-portion size or a gender specific-portion size in the calibration study of the Multiethnic Cohort.

Nutrients by Unit	Men (*n* = 1141)			Women (*n* = 1150)		
	24HDRs	QFFQ		24HDRs	QFFQ	
		Original-PS	GS-PS		Original-PS	GS-PS
	Daily intake					
Energy (kcal)	1879.2 ± 553.2	2304.0 ± 1014.7	1886.0 ± 789.2 *	1456.1 ± 422.8	1877.2 ± 866.2	1501.4 ± 634.7
Macronutrients (g/day)						
Protein	75.1 ± 24.8	85.4 ± 40.2	70.5 ± 32.6	57.7 ± 18.3	71.6 ± 34.3	56.6 ± 25.3 *
Fat	68.4 ± 28.1	78.2 ± 42.0	64.9 ± 33.1	53.2 ± 22.9	63.3 ± 35.9	50.1 ± 26.0
Saturated fat	20.6 ± 10.0	22.9 ± 13.4	19.0 ± 10.7	16.0 ± 7.7	18.4 ± 11.1	14.6 ± 8.3
Carbohydrate	233.4 ± 77.8	302.6 ± 134.7	244.4 ± 100.2	188.0 ± 58.9	260.1 ± 125.0	209.7 ± 93.1
Dietary fiber	16.8 ± 9.0	25.6 ± 14.3	21.0 ± 11.6	14.2 ± 6.5	24.3 ± 14.0	19.4 ± 10.6
Vitamins and minerals (mg/day)						
Vitamin A (IU)	7032.0 ± 6010.3	12,343.7 ± 9860.6	9222.6 ± 6166.1	6885.9 ± 5826.1	13,241.7 ± 9933.6	9491.9 ± 6154.5
Vitamin D (IU)	127.2 ± 123.4	150.8 ± 114.1	129.9 ± 94.8 *	104.2 ± 102.9	137.0 ± 102.1	113.2 ± 80.5
Vitamin E	8.8 ± 4.7	12.8 ± 8.2	10.9 ± 7.4	7.3 ± 3.8	10.6 ± 6.6	8.8 ± 5.9
Vitamin C	120.1 ± 86.5	181.7 ± 124.3	156.6 ± 101.6	113.0 ± 75.9	188.7 ± 128.7	161.3 ± 106.8
Vitamin B_6_	1.8 ± 0.8	2.5 ± 1.3	2.1 ± 1.1	1.4 ± 0.6	2.1 ± 1.1	1.7 ± 0.9
Vitamin B_12_ (mcg)	4.4 ± 5.2	5.2 ± 3.8	4.6 ± 3.5 *	3.2 ± 3.3	4.1 ± 2.8	3.5 ± 2.5
Calcium	592.3 ± 323.8	803.3 ± 426.6	674.5 ± 348.0	503.9 ± 264.1	750.8 ± 420.0	608.0 ± 332.7
Phosphorous	1113.8 ± 396.4	1382.6 ± 625.7	1145.1 ± 511.9	876.2 ± 308.8	1198.7 ± 575.8	956.0 ± 440.6
Magnesium	280.3 ± 109.1	370.4 ± 167.0	308.7 ± 136.4	221.9 ± 82.6	327.5 ± 156.1	264.2 ± 120.8
Iron	13.2 ± 5.8	18.3 ± 9.8	15.4 ± 8.6	10.3 ± 3.9	15.3 ± 8.3	12.5 ± 6.9
	Nutrient density					
% kcal						
Protein	16.0 ± 3.4	14.7 ± 2.8	14.7 ± 2.7	15.9 ± 3.4	15.0 ± 2.9	14.8 ± 2.7
Fat	31.6 ± 7.5	29.7 ± 7.0	30.1 ± 6.9	31.3 ± 7.7	29.2 ± 7.0	29.0 ± 6.8
Saturated fat	9.5 ± 3.0	8.7 ± 2.6	8.8 ± 2.6	9.4 ± 3.1	8.5 ± 2.6	8.4 ± 2.5
Carbohydrate	49.7 ± 9.1	52.1 ± 9.3	51.5 ± 8.9	51.8 ± 8.9	54.4 ± 8.8	54.7 ± 8.5
mg/1000 kcal						
Dietary fiber (g)	9.0 ± 4.0	11.1 ± 4.2	11.0 ± 4.0	10.0 ± 4.1	12.9 ± 4.5	12.7 ± 4.2
Vitamin A (IU)	3896.5 ± 3516.3	5482.8 ± 4720.2	4889.9 ± 3133.5	4969.5 ± 4519.8	7056.2 ± 4585.8	6273.4 ± 3422
Vitamin D (IU)	68.7 ± 64.5	66.1 ± 42.5 *	68.5 ± 41.8 *	72.5 ± 70.4	72.1 ± 45.5 *	73.6 ± 43.3 *
Vitamin E	4.6 ± 2.0	5.5 ± 3.1	5.8 ± 3.5	4.8 ± 2.0	5.5 ± 2.3	5.7 ± 2.8
Vitamin C	65.9 ± 44.8	80.2 ± 47.3	83.1 ± 45.9	81.4 ± 56.7	101.6 ± 56.2	106.7 ± 57.7
Vitamin B_6_	1.0 ± 0.3	1.1 ± 0.4	1.1 ± 0.4	1.0 ± 0.4	1.1 ± 0.4	1.1 ± 0.4
Vitamin B_12_ (mcg)	2.3 ± 2.5	2.3 ± 1.4 *	2.4 ± 1.5 *	2.2 ± 2.3	2.1 ± 1.1 *	2.3 ± 1.3 *
Calcium	315.6 ± 143.1	349.5 ± 128.4	354.6 ± 123.4	344.9 ± 149.7	393.8 ± 139.0	395.2 ± 134.1
Phosphorous	591.7 ± 130.9	596.6 ± 120.3 *	599.6 ± 119.5	600.5 ± 139.5	628.8 ± 127.8	624.0 ± 126.0
Magnesium	149.8 ± 40.0	160.9 ± 37.9	162.7 ± 37.7	154.9 ± 43.9	173.5 ± 39.6	174.0 ± 39.3
Iron	7.1 ± 2.5	8.0 ± 3.2	8.1 ± 3.5	7.2 ± 2.4	8.1 ± 2.6	8.1 ± 2.8

* All intake levels from the QFFQs were significantly different from reference method (*p* < 0.05 by paired t-test), except for those with a footnote.

**Table 5:** Pearson correlation coefficients among daily nutrient intakes from 24 h dietary recalls and those from quantitative food frequency questionnaire using either an original-portion size (original-PS) or a gender specific-portion size (GS-PS) in the calibration study of the Multiethnic Cohort ^1^.

	Daily Intake					Nutrient Density			
	Men (*n* = 1141)		Women (*n* = 1150)			Men (*n* = 1141)		Women (*n* = 1150)	
	Original-PS	GS-PS	Original-PS	GS-PS		Original-PS	GS-PS	Original-PS	GS-PS
Energy (kcal)	0.30	0.24	0.28	0.23					
Macronutrients (g/day)					Macronutrients (% energy)				
Protein	0.29	0.23	0.29	0.26	Protein	0.38	0.38	0.49	0.50
Fat	0.37	0.33	0.34	0.29	Fat	0.62	0.63	0.62	0.61
Saturated fat	0.45	0.42	0.42	0.39	Saturated fat	0.68	0.69	0.70	0.69
Carbohydrate	0.37	0.33	0.36	0.34	Carbohydrate	0.66	0.68	0.66	0.65
Dietary fiber	0.47	0.48	0.49	0.49	Dietary fiber (g/1000 kcal)	0.74	0.75	0.75	0.75
Vitamins and Minerals (mg/day)					Vitamins and Minerals (mg/1000 kcal)				
Vitamin A (IU)	0.45	0.43	0.47	0.46	Vitamin A (IU)	0.59	0.59	0.60	0.60
Vitamin D (IU)	0.52	0.52	0.56	0.55	Vitamin D (IU)	0.60	0.59	0.63	0.62
Vitamin E	0.27	0.24	0.25	0.19	Vitamin E	0.42	0.40	0.37	0.34
Vitamin C	0.55	0.56	0.56	0.58	Vitamin C	0.66	0.66	0.65	0.66
Vitamin B_6_	0.41	0.39	0.41	0.40	Vitamin B_6_	0.64	0.63	0.58	0.58
Vitamin B_12_ (mcg)	0.34	0.31	0.36	0.33	Vitamin B_12_ (mcg)	0.41	0.40	0.43	0.41
Calcium	0.49	0.49	0.51	0.50	Calcium	0.73	0.72	0.74	0.74
Phosphorous	0.36	0.34	0.39	0.37	Phosphorous	0.69	0.69	0.67	0.67
Magnesium	0.40	0.39	0.40	0.39	Magnesium	0.71	0.71	0.66	0.67
Iron	0.40	0.38	0.34	0.31	Iron	0.62	0.60	0.56	0.54
Average	0.40	0.38	0.40	0.38	Average	0.61	0.61	0.61	0.60

^1^ All correlations were based on log transformed nutrients and were adjusted to account for within-person variability based on only three recall values rather than a large number.

## Supplementary Materials

The following are available online at https://www.mdpi.com/2072-6643/11/7/1449/s1, Table S1: Percent of participants in the lowest and the highest quintiles of intakes from the quantitative food frequency questionnaire (QFFQ) with original-portion sizes who belong to the same quintiles from the QFFQ with gender specific-portion sizes in the calibration study of the Multiethnic Cohort.

## References

[1] Willett W. (2012). Nutritional Epidemiology.

[2] Coulston A.M., Boushey C.J., Ferruzzi M.G., Delahanty L.M. (2017). Nutrition in the Prevention and Treatment of Disease.

[3] Freedman L.S., Schatzkin A., Midthune D., Kipnis V. (2011). Dealing with dietary measurement error in nutritional cohort studies. J. Natl. Cancer Inst..

[4] Beaton G.H., Milner J., Corey P., McGuire V., Cousins M., Stewart E., De Ramos M., Hewitt D., Grambsch P., Kassim N. (1979). Sources of variance in 24-h dietary recall data: Implications for nutrition study design and interpretation. Am. J. Clin. Nutr..

[5] Hankin J.H. (1986). A diet history method for research, clinical, and community use: 23rd Lenna Frances Cooper Memorial Lecture. J. Am. Diet. Assoc..

[6] Hankin J.H., Wilkens L.R. (1994). Development and validation of dietary assessment methods for culturally diverse populations. Am. J. Clin. Nutr..

[7] Park M.K., Kim D.W., Kim J., Park S., Joung H., Song W.O., Paik H.Y. (2011). Development of a dish-based, semi-quantitative FFQ for the Korean diet and cancer research using a database approach. Br. J. Nutr..

[8] Brunstrom J.M., Rogers P.J., Pothos E.M., Calitri R., Tapper K. (2008). Estimating everyday portion size using a ‘method of constant stimuli’: In a student sample, portion size is predicted by gender, dietary behaviour, and hunger, but not BMI. Appetite.

[9] Cavazza N., Guidetti M., Butera F. (2017). Portion size tells who I am, food type tells who you are: Specific functions of amount and type of food in same-and opposite-sex dyadic eating contexts. Appetite.

[10] Kelly M.T., Rennie K.L., Wallace J.M., Robson P.J., Welch R.W., Hannon-Fletcher M.P., Livingstone M.B.E. (2009). Associations between the portion sizes of food groups consumed and measures of adiposity in the british national diet and nutrition survey. Br. J. Nutr..

[11] Lim E., Sim A., Forde C., Cheon B. (2018). The role of perceived stress and gender on portion selection patterns. Physiol. Behav..

[12] Mitchell D., Jonnalagadda S., Clutter D., Smiciklas-Wright H., Kris-Etherton P. (1995). Comparison of different methods for estimating food portion size: Gender differences. J. Am. Diet. Assoc..

[13] O’Brien S., McNulty B., Nugent A., Gibney E., Livingstone M. (2011). A comparison of gender differences in food portion sizes consumed by Irish adults during 1997 and 1999. Proc. Nutr. Soc..

[14] Lee H., Kang M., Song W.O., Shim J.E., Paik H.Y. (2016). Gender analysis in the development and validation of ffq: A systematic review. Br. J. Nutr..

[15] Kolonel L.N., Henderson B.E., Hankin J.H., Nomura A.M., Wilkens L.R., Pike M.C., Stram D.O., Monroe K.R., Earle M.E., Nagamine F.S. (2000). A Multiethnic Cohort in Hawaii and Los Angeles: Baseline characteristics. Am. J. Epidemiol..

[16] Hankin J.H., Wilkens L.R., Kolonel L.N., Yoshizawa C.N. (1991). Validation of a quantitative diet history method in Hawaii. Am. J. Epidemiol..

[17] Kang M., Park S.-Y., Boushey C.J., Wilkens L.R., Monroe K.R., Le Marchand L., Kolonel L.N., Murphy S.P., Paik H.Y. (2018). Portion sizes from 24 h dietary recalls differed by sex among those who selected the same portion size category on a food frequency questionnaire. J. Acad. Nutr. Diet..

[18] Kang M., Park S.-Y., Boushey C.J., Wilkens L.R., Le Marchand L., Kolonel L.N., Murphy S.P., Paik H.Y. (2019). Ratios of food amounts across three portion size categories on a food frequency questionnaire in men and women. J. Acad. Nutr. Diet..

[19] Stram D.O., Hankin J.H., Wilkens L.R., Pike M.C., Monroe K.R., Park S., Henderson B.E., Nomura A.M., Earle M.E., Nagamine F.S. (2000). Calibration of the dietary questionnaire for a Multiethnic Cohort in Hawaii and Los Angeles. Am. J. Epidemiol..

[20] Murphy S.P. (2002). Unique nutrition support for research at the cancer research center of Hawaii. Hawaii Med. J..

[21] (2013). SAS Statistical Software.

[22] Noöthlings U., Hoffmann K., Bergmann M.M., Boeing H. (2007). Fitting portion sizes in a self-administered food frequency questionnaire. J. Nutr..

[23] Dehghan M., Mente A., Zhang X., Swaminathan S., Li W., Mohan V., Iqbal R., Kumar R., Wentzel-Viljoen E., Rosengren A. (2017). Associations of fats and carbohydrate intake with cardiovascular disease and mortality in 18 countries from five continents (PURE): A prospective cohort study. Lancet.

[24] Joshipura K.J., Hu F.B., Manson J.E., Stampfer M.J., Rimm E.B., Speizer F.E., Colditz G., Ascherio A., Rosner B., Spiegelman D. (2001). The effect of fruit and vegetable intake on risk for coronary heart disease. Ann. Intern. Med..

[25] Park S.-Y., Wilkens L.R., Kolonel L.N., Henderson B.E., Le Marchand L. (2016). Inverse associations of dietary fiber and menopausal hormone therapy with colorectal cancer risk in the Multiethnic Cohort Study. Int. J. Cancer.

[26] Park S.-Y., Kolonel L.N., Henderson B.E., Wilkens L.R. (2012). Dietary fat and breast cancer in postmenopausal women according to ethnicity and hormone receptor status: The Multiethnic Cohort Study. Cancer Prev. Res..

[27] Sinha R., Cross A.J., Graubard B.I., Leitzmann M.F., Schatzkin A. (2009). Meat intake and mortality: A prospective study of over half a million people. Arch. Intern. Med..

[28] Tsugane S., Sasazuki S., Kobayashi M., Sasaki S. (2004). Salt and salted food intake and subsequent risk of gastric cancer among middle-aged Japanese men and women. Br. J. Cancer.

[29] Beaton G.H. (1994). Approaches to analysis of dietary data: Relationship between planned analyses and choice of methodology. Am. J. Clin. Nutr..

