# Supplementary material for: Considering Gender Differences in Portion Sizes to Improve the Accuracy of Nutrient Intakes from A Food Frequency Questionnaire
Source: Nutrients. 2019 Jun 26;11(7):1449. doi: 10.3390/nu11071449 (PMC6682990; doi:10.3390/nu11071449)
Supplement: Supplementary file 1 [file nutrients-11-01449-s001.pdf]

nutrients

MDPI

Supplementary Table S1. Percent of participants in the lowest and the highest quintiles of intakes from the quantitative food frequency questionnaire (QFFQ) with original-portion sizes who belong to the same quintiles from the QFFQ with gender specific-portion sizes in the calibration study of the Multiethnic Cohort.

Daily intake					Nutrient Density				
	Men (n = 1,141)		Women (n = 1,150)			Men (n = 1,141)		Women (n = 1,150)	
Nutrients	Lowest quintile	Highest quintile	Lowest quintile	Highest quintile	Nutrients	Lowest quintile	Highest quintile	Lowest quintile	Highest quintile
Energy (kcal)	86.0	86.0	87.0	83.9					
Macronutrients (g/day)					Macronutrients (% Calorie)				
Protein	85.1	86.0	85.2	85.2	Protein	86.4	86.4	87.0	86.1
Fat	85.5	89.0	85.7	87.0	Fat	84.2	87.3	85.7	87.4
Saturated fat	90.4	89.5	87.0	89.1	Saturated fat	86.4	89.9	87.8	91.7
Carbohydrate	84.2	80.7	86.1	84.8	Carbohydrate	89.0	85.5	87.0	84.8
Dietary fiber	89.0	90.4	84.8	87.8	Dietary fiber (g/1,000 kcal)	89.0	90.8	90.4	90.0
Vitamins and Minerals (mg/day)					Vitamins and Minerals (mg/1,000kcal)				
Vitamin A (IU)	92.1	87.3	87.4	84.3	Vitamin A (IU)	90.4	89.0	87.4	83.5
Vitamin D (IU)	91.2	90.4	93.5	88.3	Vitamin D (IU)	88.2	90.4	90.4	87.8
Vitamin E	87.7	87.7	84.3	86.1	Vitamin E	86.0	90.4	83.0	88.3
Vitamin C	91.7	86.4	90.0	90.9	Vitamin C	89.9	88.2	87.8	88.3
Vitamin B_6	87.3	88.6	87.0	86.5	Vitamin B_6	88.2	90.4	87.4	90.0
Vitamin B_{12} (mcg)	89.0	91.2	89.1	89.1	Vitamin B_{12}	89.5	9.34	86.1	89.1
Calcium	88.6	89.9	89.6	87.0	Calcium	91.7	90.4	90.9	90.9
Phosphorous	86.4	87.3	85.7	84.3	Phosphorous	87.3	89.0	88.3	90.0
Magnesium	88.2	88.6	87.0	86.1	Magnesium	89.9	90.8	87.8	90.4
Iron	86.0	89.0	87.0	86.5	Iron	88.6	90.8	90.4	89.6
Average	88.0	88.0	87.3	86.7	Average	88.3	89.5	87.8	88.5
